# circHIPK3 regulates cell proliferation and migration by sponging miR-124 and regulating AQP3 expression in hepatocellular carcinoma

**DOI:** 10.1038/s41419-017-0204-3

**Published:** 2018-02-07

**Authors:** Genwen Chen, Yanting Shi, Mengmeng Liu, Jianyong Sun

**Affiliations:** 1 Department of Gastroenterology, Zhongshan Hospital, Fudan University, Shanghai, 200032 PR China; 2 Department of Nephrology, Zhongshan Hospital, Fudan University, Shanghai, 200032 PR China

## Abstract

Noncoding RNAs plays an important role in hepatocellular carcinoma (HCC). Here, we show that miR-124 was downregulated in HCC tissues and that the ectopic expression of miR-124 inhibited the proliferation and migration of HCC cells. We proposed that aquaporin 3 (AQP3) is a direct target of miR-124. AQP3 was upregulated in HCC tissues and inversely correlated with miR-124 expression. The overexpression of miR-124 decreased AQP3 expression. Indeed, AQP3 overexpression promoted cell proliferation and migration, whereas miR-124 knockdown suppressed cell proliferation and migration. Furthermore, we found that circular RNA HIPK3 (circHIPK3) acted as a miR-124 sponge and regulated the expression of the miR-124 target gene AQP3. circHIPK3 was upregulated in HCC tissues and positively correlated with AQP3 expression. Thus, silencing circHIPK3 inhibited cell proliferation and migration by downregulating AQP3 expression. Moreover, miR-124 inhibition rescued circHIPK3 knockdown induced reduction in cell proliferation and migration, as well as AQP3 expression. *In vivo* experiments also confirmed that circHIPK3 regulated xenograft tumor growth via the miR-124-AQP3 axis. These observations indicate a possible novel therapeutic strategy involving circular RNAs in HCC.

## Introduction

Hepatocellular carcinoma (HCC) is the fifth leading and the second-most lethal carcinoma worldwide^1^. Notably, HCC is one of the most fatal carcinomas in China because of the high prevalence of hepatitis B virus (HBV) infection and high incidence of liver cirrhosis^2^. Although critical factors with important roles in HCC incidence and development have been identified, the survival rate of HCC patients has not substantially improved in the past few years. Thus, the identification of key molecular mechanisms is urgently needed for HCC. Based on this perspective, we conducted the present study.

Aquaporins (AQPs) are a family of transmembrane channels that transport water and glycerol^3,4^. Recent studies have demonstrated the important role of AQPs in tumorigenesis and cancer progression^5,6^. Aquarium 3 (AQP3) is overexpressed in HCC, and high levels of AQP3 in patients predict poor prognosis^7^. However, little is known about the role of AQP3 in HCC.

MicroRNAs (miRNAs) are important noncoding RNAs that functions by binding with the 3’-UTR of mRNAs and thereby regulating the expression of protein-coding genes^8^. The importance of miRNAs in tumor biology has been widely recognized. miR-21 promotes the proliferation, migration, and invasion of HCC cells by targeting PTEN^9^. miR-34 acts as an oncogene and its suppression is a novel anti-cancer strategy for lung cancer^10^. Previous studies have identified that miR-124 is involved and modulates some cellular phenotypes in HCC by targeting ROCK2, EZH2, or CASC3^11–13^. However, the mechanism still remains to be revealed then we propose that miR-124 may modulate HCC progression through any other ways.

In the past decades, noncoding RNAs were regarded as transcriptional noise^14^. However, novel roles of noncoding RNAs, especially circular RNA (circRNAs), have widely emerged in recent years^15,16^. Circular RNAs form circular structures through the joining of 3’ and 5’ terminals^16^. Important role of circRNAs in cancers are beginning to emerge^17–19^. circRNAs are highly stable and are resistant to RNase R-mediated degradation^20^. In light of these findings, circRNAs may be used as promising cancer markers. Mounting evidence demonstrates that circRNAs serve as miRNA sponges, thus modulating the repression of miRNA targets^21^. circMTO1 is downregulated in HCC, and low expression of circMTO1 indicates shortened survival. Knockdown of the miR-9 sponge circMTO1 promotes tumor growth by enabling miR-9-dependent downregulation of p21^22^. circPVT1 is upregulated in gastric cancer (GC), and promotes cell proliferation by acting as a sponge for the miR-125 family^23^. It is worth noting that circRNAs may thus be novel regulators in cancer^21^. However, studies investigating the expression, correlation and roles of circRNAs, miRNAs, and targets are lacking.

In the current study, we found that miR-124-3p (miR-124) was significantly downregulated in HCC and inhibited the proliferation and migration of HCC cells. Furthermore, we found that miR-124 could mediate the proliferation and migration of HCC cells by targeting AQP3. This is the first study to identify that AQP3 is a direct target of miR-124. Considering the novel function of circRNAs in cancer biology, we proposed that the downregulation of miR-124 may be mediated by circRNAs^24^. We further identified circHIPK3 as being upregulated in HCC and showed that it could promote cell proliferation and migration through AQP3 by sponging miR-124. The present study is the first to provide evidence regarding the interactions among miR-124, AQP3, and circHIPK3 in HCC, thereby identifying the potential of this axis for cancer treatment.

## Results

### miR-124 was downregulated in HCC and inhibited the proliferation and migration of HCC cells

**Fig. 1:**
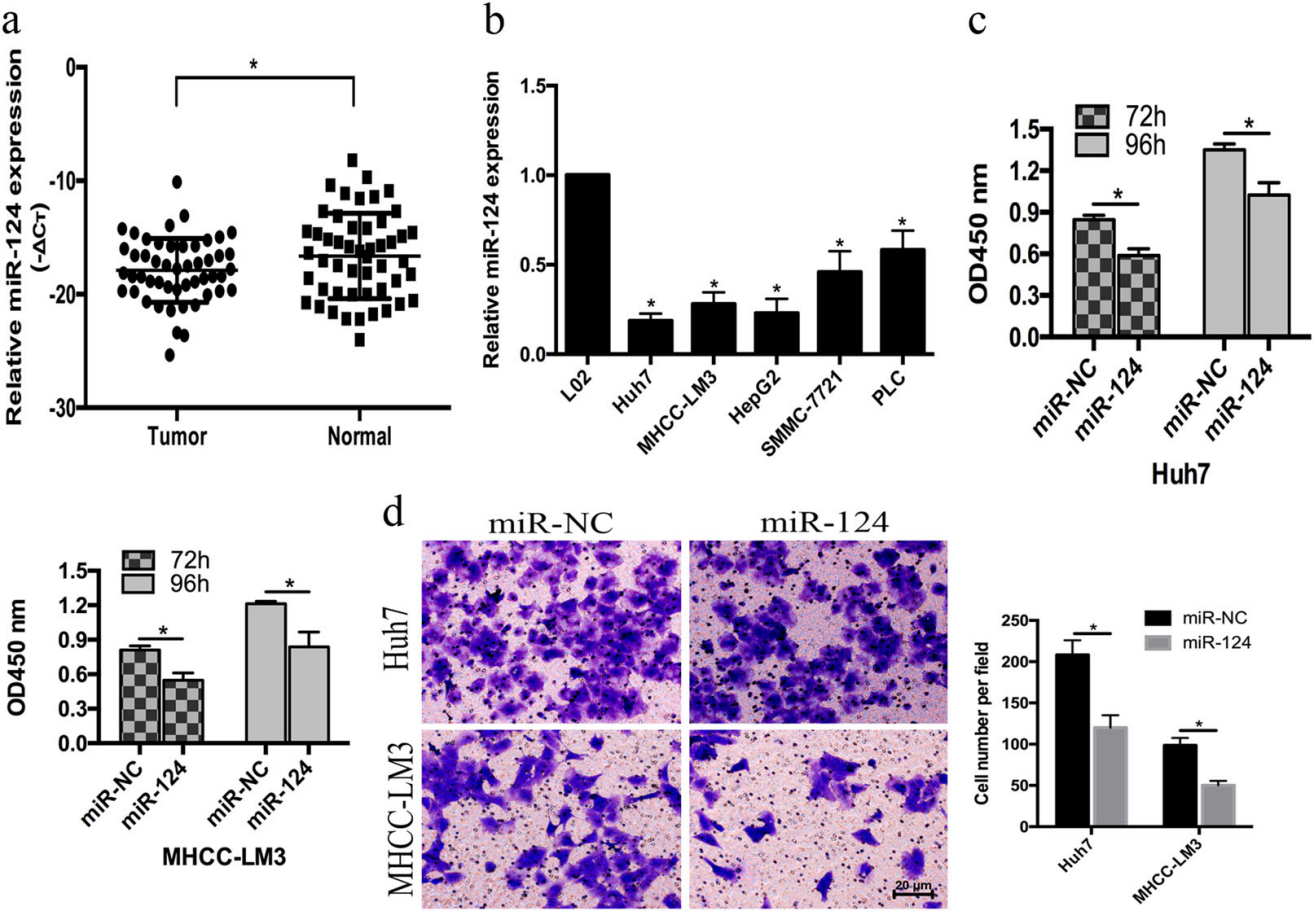
miR-124 was downregulated in HCC and inhibited the proliferation and migration of HCC cells. **a** Expression levels of miR-124 in HCC tissues and adjacent normal tissues (*n* = 50). **b** Expression levels of miR-124 in HCC and L02 cell lines. **c** CCK-8 assay after 72 h and 96 h treatment with miR-124 mimic in Huh7 and MHCC-LM3 cells. **d** The migration ability of Huh7 and MHCC-LM3 cells were measured by transwell assay (original magnification, ×200). Error bars represent mean ± SD from three independent experiments. *p < 0.05

To determine the expression of miR-124 in HCC, we analyzed miR-124 levels by qRT-PCR and found that miR-124 was significantly downregulated in HCC tissues compared with that in adjacent tissues (Fig. 1a). The results from 50 HCC patients found that miR-124 was downregulated in 36 (72%) tissues relative to adjacent tissues (*p* < 0.05). The lower expression of miR-124 was correlated with the Tumour, Node, Metastases (TNM) stage (*p* = 0.01), Barcelona Clinic Liver Cancer (BCLC) stage (*p* = 0.024) and the presence of liver cirrhosis (*p* < 0.001) (Table 1).

**Table 1:** Correlations between the clinical pathological features and expressions of miR-124, AQP3, circHIPK3, and mRNA of HIPK3

Parameter		miR-124 expression		*p*-value	AQP3 expression		*p*-value	circHIPK3 expression		*p*-value	mHIPK3 expression		*p*-value
	*n*	T > N (14)	T < N (36)		T > N (41)	T < N (9)		T > N (41)	T < N (9)		T > N (42)	T < N (8)	
*Age (year)*													
<60	32	11	21	0.181	24	8	0.182	26	6	1	26	6	0.76
≥60	18	3	15		17	1		15	3		16	2	
Sex													
Male	38	13	25	0.17	31	7	1	31	7	1	30	8	0.173
Female	12	1	11		10	2		10	2		12	0	
*Tumor size (cm)*													
≤5	20	3	17	0.095	18	2	0.409	18	2	0.409	18	2	0.581
>5	30	11	19		23	7		23	7		24	6	
*Differentiation grade*													
Well-moderate	23	6	17	0.781	22	1	0.028*	22	1	0.028*	20	3	0.883
Poor-undifferentiation	27	8	19		19	8		19	8		22	5	
*TNM stage*													
I–II	20	11	9	0.01*	13	7	0.029*	13	7	0.029*	14	6	0.07
III–IV	30	3	27		28	2		28	2		28	2	
*HBsAg*													
Negative	9	1	8	0.403	9	0	0.183	8	1	0.908	8	1	1
Positive	41	13	28		32	9		33	8		34	7	
*HBV-DNA*													
≥10^3^/copies	23	6	17	0.781	18	5	0.79	15	8	0.013*	18	5	0.526
<10^3^/copies	27	8	19		23	4		26	1		24	3	
*Cirrhosis*													
Negative	16	11	5	<0.001*	10	6	0.039*	9	7	0.04*	10	6	0.015*
Positive	34	3	31		31	3		32	2		32	2	
*GVI*													
Negative	43	10	33	0.162	38	5	0.017*	35	8	1	37	6	0.673
Positive	7	4	3		3	4		6	1		5	2	
*BCLC stage*													
A–B	42	9	33	0.024*	38	4	0.02*	35	7	0.952	36	6	0.817
C–D	8	5	3		3	5		6	2		6	2	

Given the elevated expression of miR-124 in HCC tissues, we explored miR-124 expression in HCC cell lines (Huh7, MHCC-LM3, HepG2, SMMC-7721, and PLC) and the hepatocyte cell line HL-7702 (L02) and found that miR-124 was downregulated in HCC cells (Fig. 1b). To observe the functions of miR-124 in HCC cells, we overexpressed miR-124 in Huh7 and MHCC-LM3 cells. The proliferation of Huh7 and MHCC-LM3 cells was significantly depressed 72 h and 96 h after transfection with miR-124 mimic (Fig. 1c). Meanwhile, the migration of miR-124 mimic-transfected Huh7 and MHCC-LM3 cells was also decreased compared with that of the control cells (Fig. 1d).

### AQP3 was a target of miR-124

**Fig. 2:**
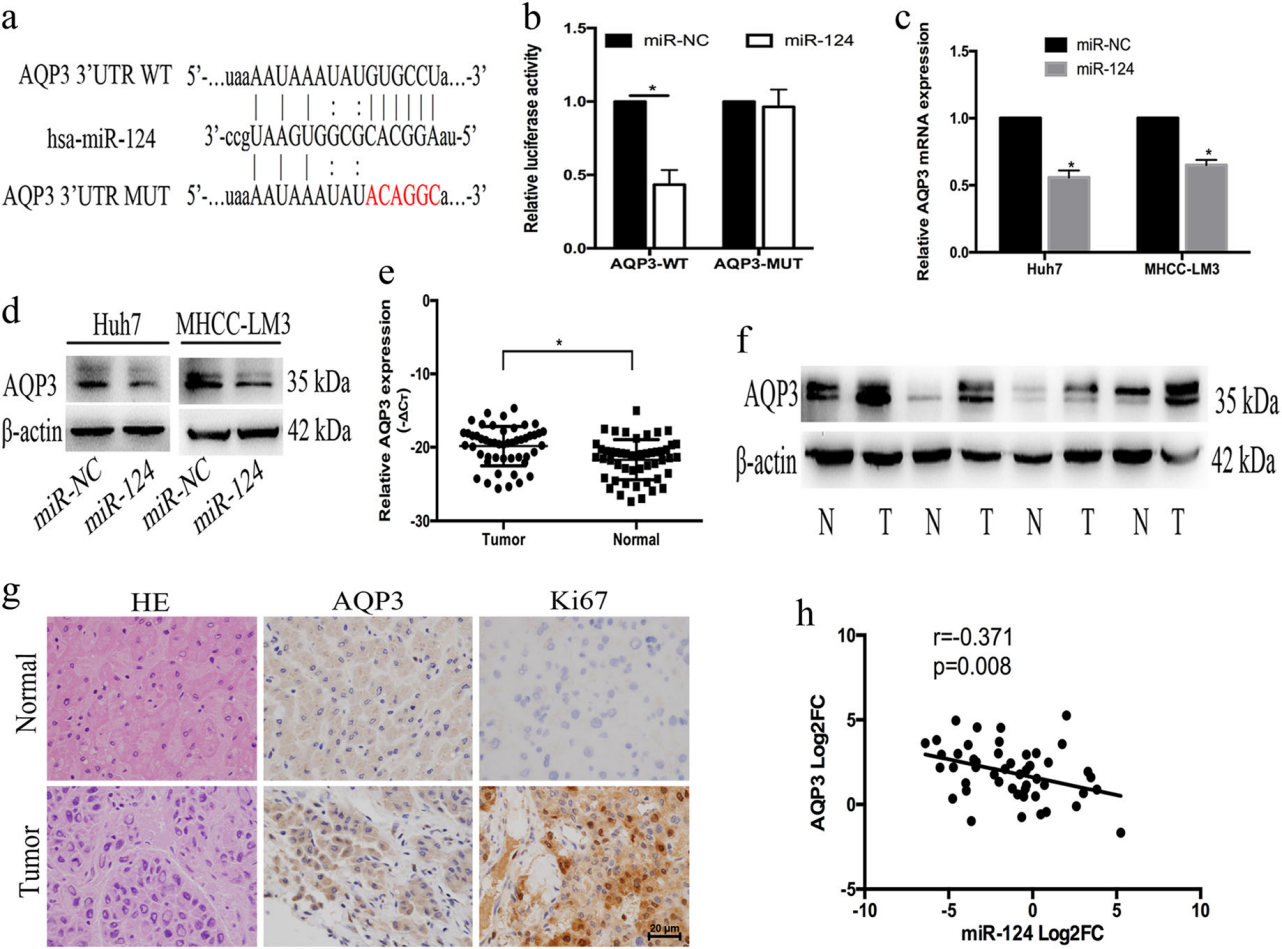
AQP3 was a target of miR-124 and was upregulated in HCC. **a** WT and MUT sequences designed for AQP3 according to the binding site for miR-124. **b** Relative luciferase activities after cotransfection pmirGLO-AQP3-WT or pmirGLO-AQP3-MUT with miR-124 mimic, respectively. **c** The mRNA expression of AQP3 was examined in Huh7 and MHCC-LM3 cells after transfection with miR-124 mimic. **d** The protein expression of AQP3 was examined by western blot in Huh7 and MHCC-LM3 cells after transfection with miR-124 mimic. **e** The AQP3 expression levels in HCC tissues and adjacent normal tissues by qRT-PCR (*n* = 50). **f** High expression of AQP3 was observed in HCC tissues by western blot. **g** Haematoxylin and eosin (H&E) and the expressions of AQP3 and Ki67 in HCC tissues and adjacent normal tissues were examined by IHC. **h** The inverse relationship between miR-124 and AQP3 in HCC tissues. Error bars represent mean ± SD from three independent experiments. *p < 0.05

To investigate the role of miR-124 in HCC, we searched miRanda database to predict miR-124 targets. As shown in Fig. 2a, the 3’-UTR of AQP3 contains a putative binding motif of miR-124. Because of important roles of AQP3 in HCC, we focused on investigating the correlation between miR-124 and AQP3. Then, luciferase reporter assay was carried out to validate whether AQP3 is a target of miR-124. We cloned wild-type (WT) and mutant 3’-UTR of AQP3 into pmirGLO plasmids, respectively. pmirGLO-AQP3-WT and pmirGLO-AQP3-MUT were cotransfected into HEK293T cells with miR-124 mimic. The luciferase activity of pmirGLO-AQP3-WT was significantly lower than that of pmirGLO-AQP3-MUT (Fig. 2b). Furthermore, qRT-PCR and western blot confirmed that the overexpression of miR-124 reduced AQP3 expression (Fig. 2c,d).

### AQP3 was upregulated in HCC and promoted the proliferation and migration of HCC cells

After establishing AQP3 as a target of miR-124, we examined the expression of AQP3 in 50 pairs of HCC samples by qRT-PCR. AQP3 was upregulated in 41 (82%) tissues compared with that in adjacent tissues (*p* < 0.05, Fig. 2e). Western blot and immunohistochemistry (IHC) were also confirmed the obvious upregulation of AQP3 in HCC (Fig. 2f,g). Upregulated of AQP3 was associated with tumor differentiation (*p* = 0.028), TNM stage (*p* = 0.029), and BCLC stage (*p* = 0.02). Moreover, high levels of AQP3 were associated with liver cirrhosis (*p* = 0.039) and gross vascular invasion (GVI, *p* = 0.017) (Table 1). Indeed, the expression level of AQP3 was inversely correlated with miR-124 expression (*p* = 0.008, Fig. 2h). Therefore, the expression of AQP3 and its correlation with malignant phenotypes also validated that AQP3 was a target of miR-124 in HCC.

**Fig. 3:**
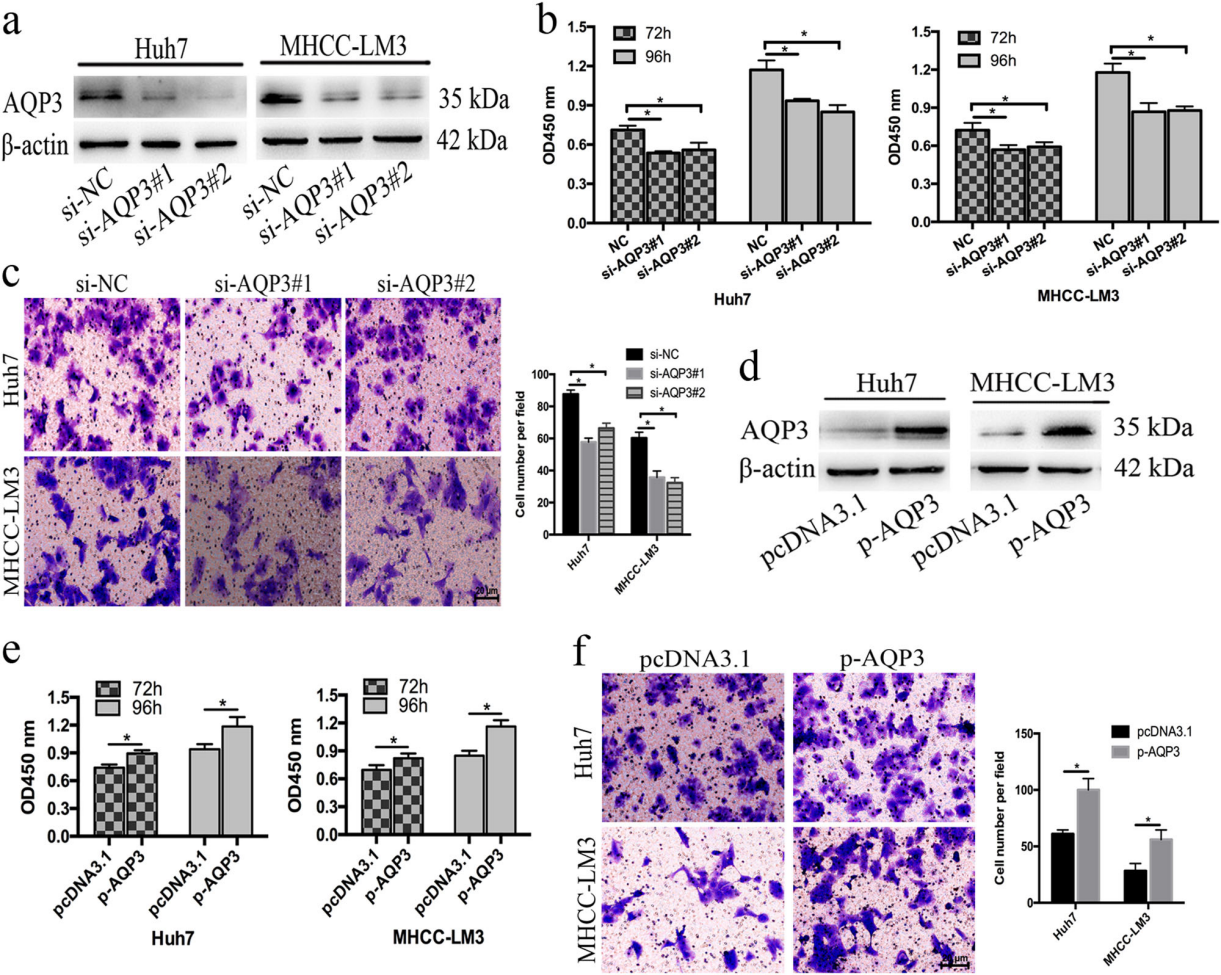
AQP3 promoted the proliferation and migration of HCC cells. **a** Knockdown efficacy of AQP3 in Huh7 and MHCC-LM3 cells by western blot analysis. **b**, **c** Knockdown of AQP3 suppressed the proliferation and migration in Huh7 and MHCC-LM3 cells determined by CCK-8 assay and transwell assay. **d** Overexpression efficacy of AQP3 in Huh7 and MHCC-LM3 cells by western blot. **e**, **f** AQP3 overexpression promoted the proliferation and migration in Huh7 and MHCC-LM3 cells by CCK-8 assay and transwell assay. Error bars represent mean ± SD from three independent experiments. *p < 0.05

We next knocked down AQP3 by siRNA in Huh7 and MHCC-LM3 cells (Fig. 3a) and found that silencing AQP3 reduced cell proliferation and migration (Fig. 3b,c). In addition, the overexpression of AQP3 promoted the proliferation and migration of Huh7 and MHCC-LM3 cells (Fig. 3d-f). Taken together, these data suggested that miR-124 likely inhibited the proliferation and migration of HCC cells by targeting AQP3.

### Identification and characterization of circHIPK3 in HCC

**Fig. 4:**
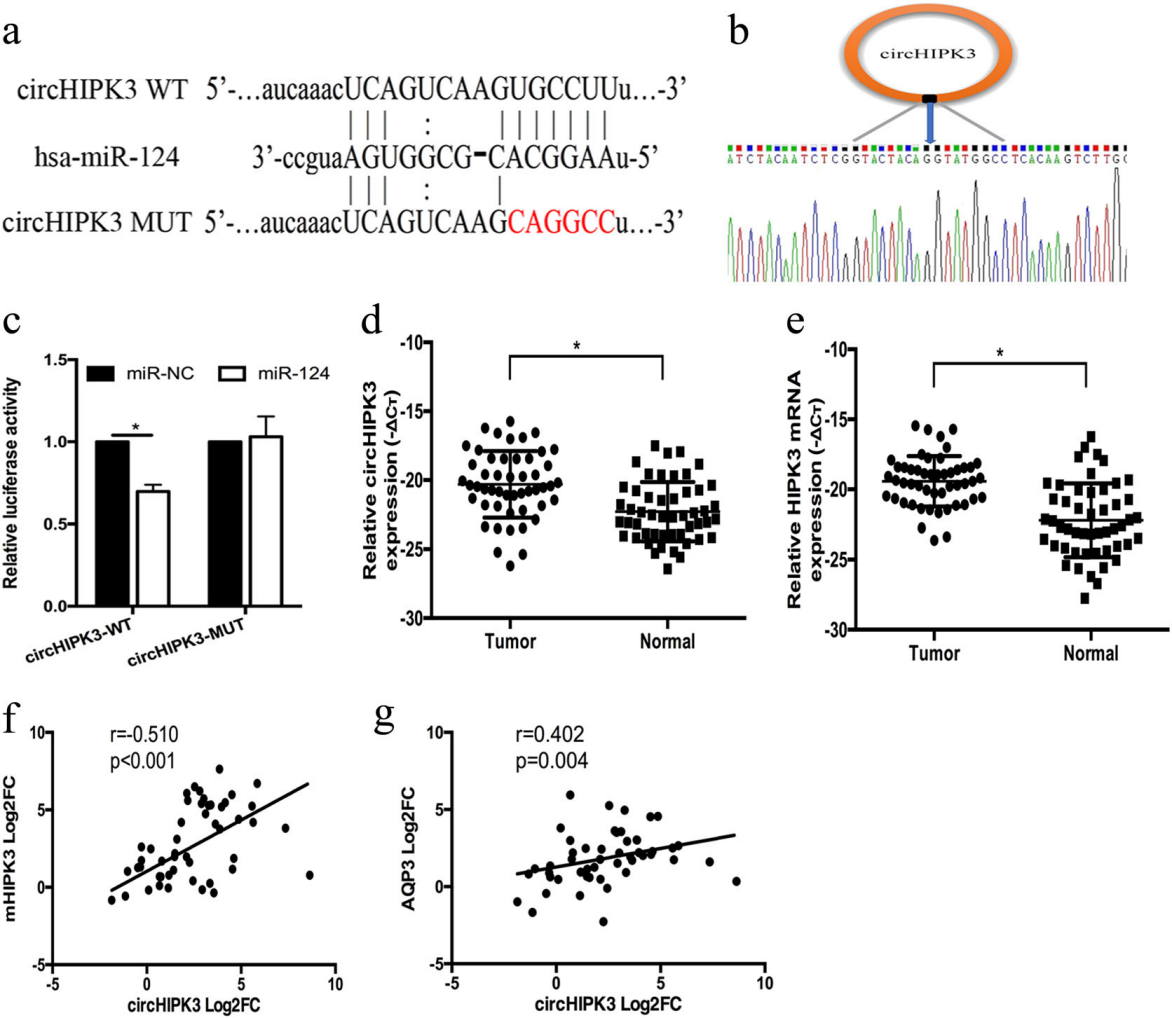
Identification and characterization of circHIPK3 in HCC. **a** Schematic representation of the target site in circHIPK3 for miR-124. **b** Schematic diagram of the head-to-tail splicing of circHIPK3 and Sanger sequencing of a PCR product. **c** Relative luciferase activities after cotransfection pmirGLO-circHIPK3-WT or pmirGLO-circHIPK3-MUT with miR-124 mimic, respectively. **d**, **e** The expression levels of circHIPK3 and HIPK3 mRNA in HCC tissues and adjacent normal tissues (*n* = 50). **f** The levels of HIPK3 mRNA were tightly correlated with circHIPK3. **g** The levels of AQP3 were positively correlated with circHIPK3. Error bars represent mean ± SD from three independent experiments. *p < 0.05

Considering the downregulation of miR-124 and the particular structure and role of circRNAs, we hypothesized that the low expression level of miR-124 may partly be attributed to the abundance of circRNAs. We identified circRNAs that might sponge with miR-124 via bioinformatics analysis based on miRanda algorithm. We found that has_circ_0000284 has a binding site for miR-124 (Fig. 4a). The circRNA has_circ_0000284, termed circHIPK3, is located in chr11:33307958-33309057 and is generated from the second exon of the HIPK3 gene^16,25^. Sanger sequencing confirmed the splice junction of circHIPK3 (Fig. 4b). Based on previous studies, circRNAs and miR-124 are predominately located in cytoplasmic^18,26^. Thus, we hypothesized that circHIPK3 may sponge with miR-124. We performed luciferase reporter assay to investigate the correlation between circHIPK3 and miR-124. Cotransfection of pmirGLO-circHIPK3-WT and miR-124 into HEK293T cells resulted in a reduction in luciferase activity compared with that from the cotransfection of pmirGLO-circHIPK3-MUT and miR-124 (Fig. 4c).

To investigate the role of circHIPK3 in HCC, we performed qRT-PCR in 50 pairs of HCC tissues. Because miR-124 was downregulated in HCC, we wondered whether miR-124 was repressed by the upregulated expression of circHIPK3 in HCC. In line with the expression of the miR-124 target AQP3, circHIPK3 was upregulated in 41 out of 50 (82%) HCC tissues compared with that in adjacent tissues (*p* < 0.05, Fig. 4d). The high expression level of circHIPK3 was correlated with tumor differentiation (*p* = 0.028) and TNM stage (*p* = 0.029). Moreover, circHIPK3 was correlated with HBV-DNA copy numbers (*p* = 0.013) and the presence of liver cirrhosis (*p* = 0.04) (Table 1). It is of note that linear mRNA of HIPK3 was upregulated in HCC tissues and HIPK3 mRNA levels were tightly correlated with circHIPK3 levels (Fig. 4e,f). However, the HIPK3 mRNA displayed no significant association with tumor differentiation, TNM stage or HBV-DNA copy number except for the presence of liver cirrhosis (*p* = 0.015). It is worth noting that the high expression level of circHIPK3 was positively correlated with the high expression of AQP3 mRNA level (*p* = 0.02, Fig. 4g), which indicated that circHIPK3 might regulate AQP3 expression by sponging miR-124.

### circHIPK3 regulated the proliferation and migration of HCC cells via the miR-124-AQP3 axis

**Fig. 5:**
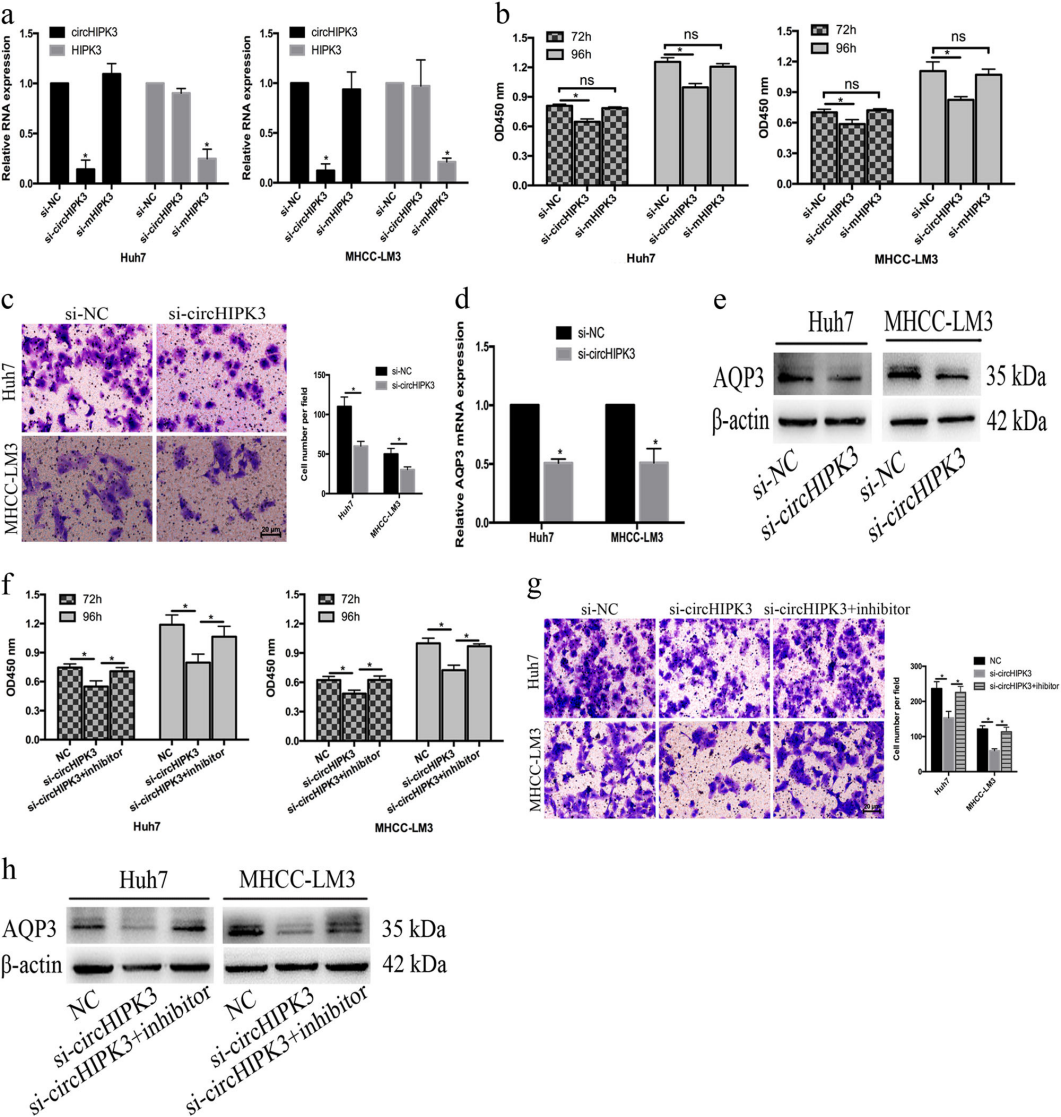
circHIPK3 regulated the proliferation and migration of HCC cells via the miR-124-AQP3 axis. **a** Knockdown efficacy of circHIPK3 and HIPK3 mRNA in Huh7 and MHCC-LM3 cells was determined by qRT-PCR, respectively. **b** The effects of circHIPK3 and HIPK3 mRNA knockdown on Huh7 and MHCC-LM3 cells by CCK-8 assay. (**c**) circHIPK3 knockdown suppressed the migration of Huh7 and MHCC-LM3 cells. **d**, **e** circHIPK3 knockdown decreased AQP3 mRNA and protein expression in Huh7 and MHCC-LM3 cells.** f**, **g** miR-124 inhibitor rescued si-circHIPK3-induced reduction of the proliferation and migration in both Huh7 and MHCC-LM3 cells. **h** Reduction of AQP3 induced by si-circHIPK3 could be rescued by miR-124 inhibition. Error bars represent mean ± SD from three independent experiments. *p < 0.05; ns: no significance

After establishing that circHIPK3 can bind with miR-124 and is positively correlated with AQP3 expression, we proposed that circHIPK3 serves the same role in HCC. We knocked down circHIPK3 via specific siRNA targeting the splice junction of circHIPK3. As expected, the expression of circHIPK3 was knocked down while HIPK3 mRNA expression was not affected (Fig. 5a). The proliferation of Huh7 and MHCC-LM3 cells was significantly repressed by si-circHIPK3, but there was no difference between cells transfected with si-mHIPK3 and siRNA negative control (Fig. 5b). Therefore, circHIPK3 silencing reduced proliferation of Huh7 and MHCC-LM3 cells (Fig. 5c).

If a circRNA acts as a miRNA sponge, its destruction could trigger downregulation of miRNA targets^24^. To investigate whether circHIPK3 regulated proliferation and migration in Huh7 and MHCC-LM3 cells by targeting AQP3 via sponging with miR-124, we silenced circHIPK3 and found that both mRNA and protein levels of AQP3 were decreased (Fig. 5d,e). However, we could not found obvious increase in miR-124 level owing to circHIPK3 knockdown. This is in line with the findings from studies on human cerebellar degeneration-related protein 1 transcript (CDR1as), where the knockdown of CDR1as affects HEK293T cells potentially through indirect miR-7 or miR-7 independent CDR1as function, whereas had no miR-7-specific effects^24^. To identify whether circHIPK3 regulates HCC cell proliferation and AQP3 expression by inhibiting miR-124, we performed rescue experiments. circHIPK3 knockdown induced a reduction in the proliferation and migration of Huh7 and MHCC-LM3 cells could be rescued by the miR-124 inhibitor (Fig. 5f,g). Furthermore, the suppression of AQP3 by circHIPK3 could also be rescued after miR-124 inhibition (Fig. 5h). These data suggested that circHIPK3 regulated the proliferation and migration of HCC cells via the miR-124-AQP3 axis.

### Knockdown of circHIPK3 suppressed tumor growth *in vivo*

**Fig. 6:**
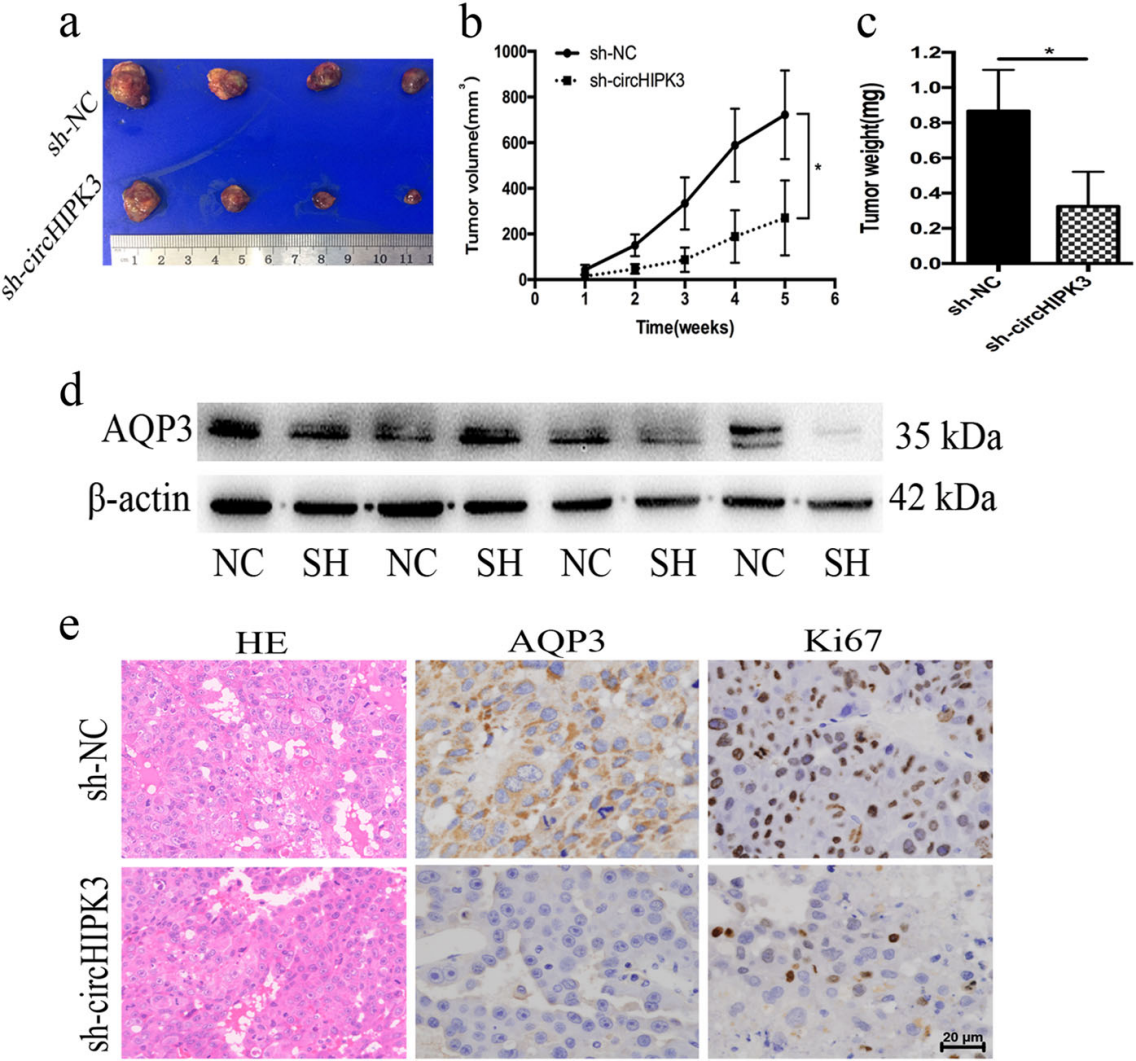
Knockdown of circHIPK3 suppressed tumor growth *in vivo*. **a** The xenograft tumors were significantly depressed by circHIPK3 depletion. **b**, **c** Tumor volume and tumor weight of the xenografts were remarkably inhibited by sh-circHIPK3. **d** circHIPK3 depletion downregulated AQP3 expression *in vivo* by western blot analysis. **e** The expressions of AQP3 and Ki67 in the xenograft tumors were examined by IHC. Error bars represent mean ± SD from three independent experiments. *p < 0.05

*In vitro* study demonstrated that circHIPK3 knockdown suppressed the viability of Huh7 and MHCC-LM3 cells. Thus, we speculated that circHIPK3 plays the same role *in vivo*. Huh7 cells with stable circHIPK3 knockdown (sh-circHIPK3-Huh7) or cells infected with control vector (sh-NC-Huh7) were injected into nude mice. Tumor volume was measured every week for 5 weeks. The mice were killed by anesthesia, and xenografts were removed for further analyses. In accordance with the *in vitro* results, circHIPK3 depletion repressed Huh7 xenograft growth *in vivo*. Proliferation marker Ki67 staining shown that the growth of sh-circHIPK3-Huh7 xenografts was reduced compared with that of sh-NC-Huh7 xenografts (Fig. 6a-c). Western blot and IHC also validated the downregulation of AQP3 in sh-circHIPK3-Huh7 xenografts *in vivo* (Fig. 6d,e). In conclusion, circHIPK3 could regulated HCC tumor growth via the miR-124-AQP3 axis.

## Discussion

miRNAs are identified as indispensable epigenetic regulators of gene expression in cancers. The role of miRNAs is multidimensional and markedly different based on the tumor biology^27^. In our present study, we identified that miR-124 was downregulated in HCC and that it inhibited the proliferation and migration of HCC cells. miR-124 has also been identified as a tumor suppressor in many cancers and has been shown to play different functional roles in various cancers^28–30^. The mechanism of miR-124 in HCC remains elusive. Canonically, miRNAs function via binding with the 3’-UTR of mRNAs, resulting in the degradation of mRNAs or translational blockage^31^. In this regard, we verified a new target of miR-124, AQP3, and its role in HCC. AQP3 is an important member of transmembrane channel, and it plays a crucial role in the transportation of water across the cell membrane. *In vitro* experiments have shown that miR-124 overexpression reduces AQP3 expression by facilitating the degradation of AQP3 mRNA. In our study, we found that AQP3 was upregulated in HCC and that AQP3 expression was closely associated with miR-124. It has been reported that high levels of AQP3 predicted poor survival^7^. We postulated that AQP3 plays crucial roles in mediating malignant phenotypes in HCC. Functional experiments revealed that the overexpression of AQP3 promoted the proliferation and migration of HCC cells, whereas silencing AQP3 reduced cell proliferation and migration, which was opposite to the effects of miR-124.

As a novel class of noncoding RNAs, accumulating evidence suggests that circRNAs may be used as tumor biomarkers and regulators^32–34^. Through the canonical pathway, circRNAs act as miRNA sponges. circLARP4 was downregulated in GC tissues and inhibited the proliferation and invasion of GC cells by sponging miR-424^35^. circRNA_100290 was upregulated in oral cancer and functioned as sponge of the miR-29 family. In addition, knockdown of circRNA_100290 inhibited cell proliferation and decreased the miR-29 target CDK6, and this effect could be rescued by the miR-29 inhibitor^36^. CDR1as was demonstrated to be abundant in neuronal tissues and to be highly co-expressed with miR-7 in these tissues. CDR1as was founded to be densely bounded by miRNA effector complexes and was shown to harbor up to 63 conserved binding sites for miR-7. However, the depletion of CDR1as had no obvious effect on miR-7 expression^24^. These results are in accordance with our findings. Accumulating studies on the role of circRNAs as competing endogenous RNA have indicated that circRNAs may serve as miR-124 sponges. Bioinformatics analysis found that circHIPK3 may sponge miR-124. circHIPK3 is a multifunctional circRNA that plays roles in retinal vascular dysfunction in diabetes, bladder cancer and including HCC. Previous study revealed that circHIPK3 was upregulated in diabetic retinas and promoted endothelial proliferation and vascular dysfunction by blocking miR-30a^37^. However, circHIPK3 was downregulated in bladder cancer and suppressed cell migration, invasion, and angiogenesis by sponging with miR-558^38^. These indicate that circHIPK3 has multifunctional roles in diseases, respectively. Indeed, the role of circHPIK3 in HCC remains unclear, thus we want to reveal the function of circHIPK3 in HCC. We carried out luciferase reporter assay and found that circHIPK3 had a binding site for miR-124. Functional experiments validated that silencing circHIPK3 inhibited the proliferation and migration of HCC cells. Notably, the HIPK3 mRNA was also upregulated in HCC and tightly correlated with circHIPK3 expression. However, silencing HIPK3 mRNA had no effect on cell proliferation. These data indicate the novel role of circRNAs in tumor biology.

If circRNAs serve as miRNA sponges, circRNAs depletion would lead to downregulation of the miRNA targets, which is in accordance with our finding that silencing circHIPK3 downregulated the miR-124 target AQP3. Furthermore, the reduction in the protein level of AQP3 and cell viability and migration could be rescued by miR-124 inhibition, which was in accordance with the function of circRNA_100290^36^. This indicated that circHIPK3 regulates AQP3 expression via miR-124. Moreover, the high expression level of circHIPK3 was positively correlated with AQP3 expression. *In vivo* study also confirmed that circHIPK3 depletion suppressed HCC tumor growth via the miR-124 target AQP3. Thus, we identified AQP3 as a miR-124 target and showed that circHIPK3 regulates AQP3 expression by sponging miR-124. Indeed, we investigated the expression levels and correlations among circHIPK3, miR-124, and AQP3 in HCC tissues and the associated mechanism by western blot, luciferase assay and immunostaining both *in vitro* and in vivo. However, the use of transgenic mice overexpressing circHIPK3 in the future may enable researchers to better simulate HCC biology. Taken together, our data revealed that circHIPK3 regulates the proliferation and migration of HCC cells via the miR-124-AQP3 axis.

Here, for the first time, we identified AQP3 as a target of miR-124 and showed that the miR-124-AQP3 axis regulates cell proliferation and migration in HCC. circHIPK3 regulates the proliferation and migration of HCC cells by serving as a miR-124 sponge and thus modulates AQP3 expression. *In vivo* study confirmed that circHIPK3 knockdown suppresses xenograft tumor growth. Further understanding of the circHIPK3-miR-124-AQP3 axis may provide a novel therapeutic strategy for HCC in the future.

## Materials and methods

### Patients and tissue specimens

Fifty patients were enrolled in this study. All the patients were pathologically diagnosed with hepatocellular carcinoma. Patients with a history of chemotherapy and radiotherapy were excluded. Samples were immediately frozen in liquid nitrogen. Clinical tissues from patients were obtained with informed consent, with approval from the Ethics Committee of Zhongshan Hospital, Fudan University.

### Cell culture

Human hepatocellular carcinoma cell lines Huh7, MHCC-LM3, HepG2, SMMC-7721, and PLC, the hepatocyte cell line L02 and HEK293T cells were obtained from the Liver Cancer Institution of Zhongshan Hospital, Fudan University. All cells were maintained at 37°C with 5% CO_2_ in Dulbecco’s Modified Eagle’s Medium (DMEM, HyClone Laboratories, Logan, UT, USA) supplemented with streptomycin (100 µg/ml), penicillin (100 µg/ml) (Gibco), and 10% fetal bovine serum (FBS, Gibco, Grand Island, NY, USA).

### Vector construction, lentivirus production, and transfections

For AQP3 overexpression, the full-length coding sequence of AQP3 was cloned into the pcDNA3.1 vector and transfected into Huh7 and MHCC-LM3 cells. The empty pcDNA3.1 vector was used as a negative control. To construct the AQP3-3’-UTR reporter plasmid, the full-length of 3’-UTR of AQP3 and the mutated sequences were synthesized and cloned into *Nhe*I and *Sal*I sites downstream of cytomegalovirus promoter-driven luciferase cassette in the pmirGLO vector (Promega, Madison, WI, USA). To construct the circHIPK3 reporter plasmid, the coding sequence of circHIPK3 and the mutated sequences were synthesized and cloned into the pmirGLO vector.

Lentiviral vector for the complementary oligonucleotides of small hairpin RNA targeting circHIPK3 were constructed by Hanyin (Shanghai, China). Empty lentiviral vector was used as negative control. To generate stable circHIPK3 knockdown cells, Huh7 cells were seeded in six-well plate and infected with corresponding lentiviruses along with 8 µg/ml of polybrene. After 24 h, 4 µg/ml of puromycin was added for selecting stable circHIPK3 knockdown Huh7 cells. siRNAs targeting the circHIPK3 splice junction, HIPK3 mRNA, and AQP3(si-AQP3#1 and si-AQP3#2), miR-124 mimic, miR-124 inhibitor and siRNA control, mimic control, and inhibitor control were all synthesized by RiboBio (Guangzhou, China). The siRNA sequences were listed in Supplementary Table 1. Lipofectamine 3000 (Invitrogen, Eugene, OR, USA) was used for all the transfections according to the manufacturer’s instructions.

### CCK-8 assay

Cell proliferation was measured using the Cell Counting Kit-8 (CCK-8, Dojindo Chemical Laboratory, Kumamoto, Japan). A total of 5 × 10^3^ cells were seeded into 96-well plates and cultured for 24 h. The cells were transfected with siRNAs, miR-124 mimic, miR-124 inhibitor, and plasmids, and then cell viability was measured using the CCK-8 solution reagent. 10 µl of CCK-8 solution was added per well, and the plate was incubated at 37°C for 2 h. Then, absorbance was measured at 450 nm.

### Transwell migration assay

A total of 2 × 10^4^ Huh7 or MHCC-LM3 cells were resuspended in 200 µl serum-free DMEM medium and seeded into the upper chambers of transwell plates (8 μm size, Corning, NY, USA) after transfection for 48 h. The lower chamber contained 600 µl DMEM medium with 10% FBS. After incubation for 24 h at 37°C in a culture incubator, the non-migrated cells were removed. Cells that had migrated to the bottom of the membrane were fixed with 4% paraformaldehyde and stained with 0.1% crystal violet and counted under a × 200 microscope (Olympus Corporation, Tokyo, Japan).

### RNA extraction and quantitative reverse transcription-polymerase chain reaction (qRT-PCR)

Total RNA was extracted from cells and tissues using TRIzol reagent (Invitrogen, Carlsbad, CA, USA). miR-124-specific stem-loop primer was used to perform reverse transcription and quantification of miR-124 was performed using Bulge-Loop miRNA qRT-PCR Starter Kit (RiboBio), and U6 was used as an endogenous reference. For the quantification of circHIPK3 and mRNAs, 1 μg RNA was reverse transcribed into cDNA using Prime Script RT reagent Kit (Takara Bio, Shiga, Japan). 18sRNA was used as an endogenous reference. qPCR was performed on an ABI Prism 7500 Fast Real-Time PCR system (Applied Biosystems, Foster City, CA, USA) using SYBR Premix Ex Taq II (TaKaRa), and 2^-∆∆Ct^ method was used to calculate the relative expression of targets. The primers used were listed in Supplementary Table 2.

### Western blot

Total protein was extracted from cells and tissues using radio immunoprecipitation assay lysis buffer. Proteins were fractionated on 10% sodium dodecyl sulphate-polyacrylamide gels and transferred to polyvinylidene difluoride membranes (Millipore Corp., Billerica, MA, USA). After blocking for 1 h at room temperature, the membranes were incubated with rabbit anti-AQP3 antibody (1:400, #AQP-003, Alomone Laboratories, Jerusalem, Israel) and mouse against β-actin antibody (1:1000, #3700, Cell Signaling Technology, Danvers, MA, USA) respectively overnight at 4°C. Then, the membranes were incubated with horseradish peroxidase (HRP)-conjugated anti-rabbit (1:2000, #7074, Cell Signaling Technology) or anti-mouse IgG (1:2000, #7076, Cell Signaling Technology) secondary antibodies at room temperature for 1 h. The brands were visualized using an enhanced chemiluminescence detection kit and brand intensity was determined with Quantity One 4.6.2 software (Bio Rad, Hercules, CA, USA).

### Luciferase reporter assay

For luciferase reporter assay, 5 × 10^5^ HEK293T cells were seeded in 24-well plates overnight. Then, 150 ng of pmirGLO-AQP3-WT or pmirGLO-circHIPK3-WT reporter plasmids and their respective mutated vectors were cotransfected into cells with 50 nM miR-124 mimic using Lipofectamine 3000. After culturing the cells for 36 h, Firefly and Renilla luciferase activities were determined using a Dual-Luciferase Reporter Assay System (Promega) according to the manufacturer’s instructions. The relative luciferase activities were calculated based on Firefly/Renilla fluorescence.

### IHC

Tissues were fixed with 4% formalin and then embedded in paraffin. Endogenous peroxidases activity was blocked, and antigen retrieval was performed after the deparaffinization and rehydration of slides. The slides were incubated overnight at 4°C with antibodies against for Ki67 (1:400, #9449, Cell Signaling Technology) and AQP3 (1:100, #SC-20811, Santa Cruz, Paso Robles, CA). Then, the slices were incubated with HRP-conjugated secondary antibodies at 37°C for 1 h. The positivity of immunoreactivity was determined by proportion of positive cells.

### *In vivo* study

Five-week-old nude mice were raised and randomly divided into two groups. sh-circHIPK3 and sh-NC Huh7 cells (10^7^) were injected into each mouse, respectively. Tumor size was measured every week by calculating the length and width of tumors (tumor volume = 1/2 length × width^2^). Five weeks later, the mice were sacrificed and the xenografts were removed. All animal studies were approved by the Animal Ethics Committee of Zhongshan Hospital, Fudan University, and animal experiments were performed following the National Institute of Health Guide for the Care and Use of Laboratory Animals.

### Statistical analysis

Data were expressed as the mean ± standard deviation (mean ± SD). Student’s *t-*test was used to analyze differences between two experimental groups. The differences among more than two groups were analyzed by one-way analysis of variance. Pearson’s correlation coefficient analysis was used to assess the correlations. The *χ*^2^-test or Fisher’s exact test was used to analyze the association of targets with clinicopathological parameters. Statistical analyses were carried out using SPSS 21.0 software, (Chicago, IL, USA), and *p *< 0.05 was used to define statistical significance.

## Electronic supplementary material

Supplementary Table
