# Supplementary material for: circHIPK3 regulates cell proliferation and migration by sponging miR-124 and regulating AQP3 expression in hepatocellular carcinoma
Source: Cell Death Dis. 2018 Feb 7;9(2):175. doi: 10.1038/s41419-017-0204-3 (PMC5833724; doi:10.1038/s41419-017-0204-3)
Supplement: Supplementary file 1 — Supplementary Table [file 41419_2017_204_MOESM1_ESM.docx]

**Table S1. siRNA sequences**

| **siRNA** | **Sequences** | |  |
| --- | --- | --- | --- |
| si-AQP3#1 | 5′- CCUUUGCCAUGUGCUUCCU -3′ |  | |
| si-AQP3#2 | 5′-GGGUCGUCACUCCUUUAAU-3′ |  | |
| si-mHIPK3 | 5′- GCUGAUUGAUGCAGAUUUA-3′ |  | |
| si-circHIPK3 | 5′-CUACAGGUAUGGCCUCACA -3′ |  | |
| si-NC | 5′- UUCUCCGAACGUGUCACGU-3′ |  | |

**Table S2. Primers for qRT-PCR**

| **Targets** | **Sequences** | |
| --- | --- | --- |
|  | **Forward** | **Reverse** |
| AQP3 | 5′- CCGTGACCTTTGCCATGTG -3′ | 5′- CGAAGTGCCAGATTGCATCATAA -3′ |
| circHIPK3 | 5′- TATGTTGGTGGATCCTGTTCGGCA -3′ | 5′- TGGTGGGTAGACCAAGACTTGTGA -3′ |
| mHIPK3 | 5′- TGGAGACTGGGGGAAGATGA -3′ | 5′- CACACTAACTGGCTGAGGGG -3′ |
| 18sRNA | 5′- ACTCAACACGGGAAACCTCA -3′ | 5′- AACCAGACAAATCGCTCCAC -3′ |
